# Indel locations are determined by template polarity in highly efficient *in vivo* CRISPR/Cas9-mediated HDR in Atlantic salmon

**DOI:** 10.1038/s41598-019-57295-w

**Published:** 2020-01-15

**Authors:** Anne Hege Straume, Erik Kjærner-Semb, Kai Ove Skaftnesmo, Hilal Güralp, Lene Kleppe, Anna Wargelius, Rolf Brudvik Edvardsen

**Affiliations:** Institute of Marine Research, P.O. Box 1870, Nordnes, NO-5817 Bergen Norway

**Keywords:** Mutagenesis, Genetic engineering

## Abstract

Precise gene editing such as CRISPR/Cas9-mediated homology directed repair (HDR) can increase our understanding of gene function and improve traits of importance for aquaculture. This fine-tuned technology has not been developed for farmed fish including Atlantic salmon. We performed knock-in (KI) of a FLAG element in the *slc45a2* gene in salmon using sense (S), anti-sense (AS) and double-stranded (ds) oligodeoxynucleotide (ODN) templates with short (24/48/84 bp) homology arms. We show *in vivo* ODN integration in almost all the gene edited animals, and demonstrate perfect HDR rates up to 27% in individual F0 embryos, much higher than reported previously in any fish. HDR efficiency was dependent on template concentration, but not homology arm length. Analysis of imperfect HDR variants suggest that repair occurs by synthesis-dependent strand annealing (SDSA), as we show for the first time in any species that indel location is dependent on template polarity. Correct ODN polarity can be used to avoid 5′-indels interrupting the reading frame of an inserted sequence and be of importance for HDR template design in general.

## Introduction

Aquaculture continues to grow faster than any other major food production sector and is quickly becoming the main source of seafood in human diets. In this context, Norway is the largest producer of farmed Atlantic salmon (*Salmo salar*) worldwide. In later years, the production of salmon in Norway has ceased to grow due to sustainability challenges linked to open sea-cage rearing. Genetic introgression of farmed salmon into wild stocks and the marine parasite, salmon louse, are recognized as the two major concerns^1^. The high prevalence of salmon lice in most Norwegian fjords, due to open sea-cage farming, cause high lethality in wild salmonids and is hindering expansion of sea-cage farming. The consequences of genetic introgression caused by escapees remain uncertain, but existing knowledge indicates that it may lead to changes in life‐history traits, with potential ecological impacts^2–5^. Sequencing of the salmon genome^6^ has permitted more detailed studies on the link between genes and key traits, and we and others have shown that single nucleotide polymorphisms (SNPs) to a certain degree can explain the time of maturity^1^ and disease resistance^7,8^. In this context, New Breeding Technologies (NBTs) by gene editing may offer a solution to some of the problems in salmon farming, with a possible production of salmon displaying traits such as disease resistance and sterility^9–12^.

We have previously demonstrated the feasibility of double allelic KO in F0 salmon using CRISPR/Cas9, by targeting genes essential for pigmentation^9^, elongation of polyunsaturated fatty acids^13^ and reproduction^10^. At the same time, CRISPR/Cas9 KO-mutations targeting various phenotypes have been shown by others in several farmed fish species such as tilapia^14–21^, sea bream^22^, sterlet^23^, channel catfish^24,25^, southern catfish^26^, common carp^27^, sturgeon^28^ and rainbow trout^29^. CRISPR/Cas9 KOs are produced by a Cas9-induced double-stranded DNA break (DSB) followed by activation of the endogenous error-prone non-homologous end joining (NHEJ) pathway, which introduce indels at the repair junction. In fish with a long generation time such as salmon (3–4 years), it is a necessity to study the KO in the F0 generation, however the mosaicism caused by NHEJ may include partly functional in-frame indels affecting the result. As such, controlled insertions utilizing the homology directed repair (HDR) mechanism instead of NHEJ, may for example be used to effectively insert a stop codon which can increase the homogeny of the KO already in F0^30^.

DSB-repair by HDR can occur by several pathways^31^ such as synthesis-dependent strand annealing (SDSA), or by the formation of Holliday junctions that can be resolved with or without crossing-over^32^. HDR occurs naturally during meiosis but can also be activated by supplying a DNA repair template homologous to the CRISPR/Cas9 target sequence, as shown from studies in cells and model organisms^33^. This approach allows incorporation of the desired genetic changes into the repair template. A functional protocol for CRISPR/Cas9-mediated HDR in salmon offers the potential to insert genes, SNPs or regulatory elements without introducing transgenes and is therefore also interesting in the context of NBT, where such technology can be useful to target for example gene expression levels or to insert stop codons. With the exception of very low efficiency KI of a gene encoding red fluorescent protein in rohu carp^34^, such advanced and fine-tuned genome editing has not been developed for farmed fish, and it may be useful to learn from protocols already established in model fish species. While only a few studies have reported HDR in medaka^35,36^, several KI-strategies have been reported in zebrafish using either donor plasmids^37–39^ or single-stranded oligodeoxynucleotides (ssODNs)^40–45^, or both^46^. Knowledge from other fish studies are somewhat inconclusive when it comes to deciding the strategy for applying HDR in salmon, as there is a lack of consensus regarding the impact of different repair templates, homology arm length and strand complementarity in the above-mentioned studies.

**Figure 1:**
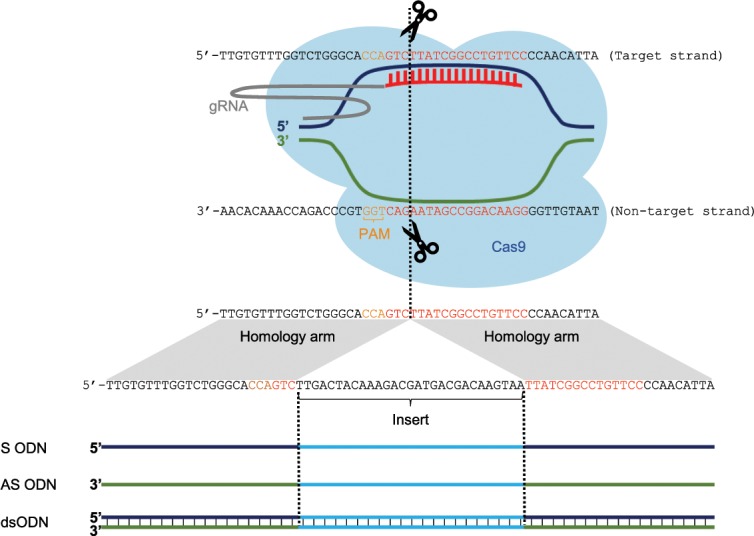
CRISPR target sequence and the donor DNA templates for the Atlantic salmon *slc45a2* gene. The ODNs were designed by copying 24/48/84 nucleotides on each side of the CRISPR cut site (indicated with a dotted line), and an insert consisting of FLAG followed by a STOP codon (TAA). In order to keep the open reading frame of FLAG, two nucleotides (TT) were added in the 5′end of the FLAG sequence. The ODN S is sense relative to the Atlantic salmon *slc45a2* gene.

Here, we aimed to establish an efficient method for controlled KI of a FLAG element in F0 salmon, targeting the pigmentation gene *solute carrier family 45 member 2* (*slc45a2*) (Fig. 1). Using next generation sequencing (NGS), we could characterize the efficiency, accuracy and types of integrations formed, which was dependent on template concentration, but not on homology arm length. Interestingly, we obtained a high level of perfect integration, up to 27%. Also, we observed that the rate of in-frame integration was higher using anti-sense (AS) as compared to sense (S) and double-stranded (ds) ODNs.

## Results

Targeting the pigmentation gene *slc45a2*, we have performed KI of a FLAG element in F0 salmon using CRISPR/Cas9 and symmetrical DNA repair templates (Fig. 1). Wierson *et al*.^39^ have shown that short homology arms from 24–48 bp is enough to direct precise and efficient knock-in in zebrafish. Based on this we have tested 24, 48 and 84 bp homology arms in this study. We also explored the effect of different templates, concentrations and polarity on the HDR efficiency.

### Identification of FLAG-positive *slc45a2-*mutants

In order to identify fish containing the FLAG insert, positive *slc45a2*-mutants were selected by visual inspection (Supplementary Fig. 1), followed by DNA extraction from fin clips and PCR (Table 1 and Supplementary Fig. 2). We were able to detect FLAG in as many as 94–100% of the mutants injected with the highest ODN concentration (1.5 µM), and in 69% of the mutants injected with the lowest concentration (0.15 µM). In contrast, FLAG was only detected in 10–12% of the mutants injected with the two different concentrations (2.5 and 10 ng/µl) of the plasmid (see Methods for description of the plasmid). Moreover, the PCR-screening did not show any difference in efficiency between the different homology arm lengths (24, 48 and 84 bp) tested in this study (Table 1).

**Table 1:** Comparison of integrate efficiency among different repair templates and concentrations, analyzed by PCR screening.

Repair template	Concentration	# samples	# FLAG positive	FLAG positive (%)
Plasmid 24	2.5 ng/µl (0.001 µM)	49	5	10
	10 ng/µl (0.004 µM)	50	6	12
S 24 ODN	1.5 µM	36	36	100
AS 24 ODN	0.15 µM	13	9	69
	1.5 µM	34	32	94
ds 24 ODN	1.5 µM	34	34	100
S 48 ODN	1.5 µM	48	48	100
S 84 ODN	1.5 µM	48	48	100

### NGS analysis of CRISPR-mutants

**Figure 2:**
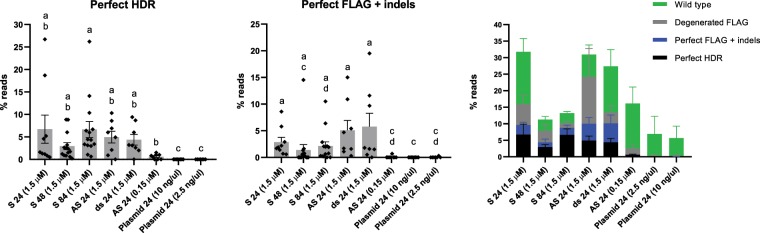
NGS results. A fragment covering the entire CRISPR target site was amplified (76 fish) prior to Illumina MiSeq sequencing. When reporting read counts with the inserted sequence, we distinguished between the following groups; **(a)** Perfect HDR (reads with a perfect match to the entire target sequence), and **(b)** Perfect FLAG + indels (reads with a correct insert sequence but mismatches/indels in the homology arms). We also reported reads with mismatches in the insert sequence (referred to as degenerated FLAG) and wild type reads (Supplementary Fig. 3). All the data are summarized in **(c)**. Read counts for each group are given in % of the total number of reads with at least 100 identical reads, for each sample. Individual samples are represented by black diamonds, and grouped for each of the different repair templates, at different concentrations (represented by grey bars). The error bars indicate the SEM of the mean for each group. Non-parametric statistics (Kruskall-Wallis) were performed to analyze the differences in HDR efficiencies between the different repair templates. Different lower-case letters indicate significant differences (*P* < 0.05).

Next, we deep sequenced 76 FLAG-positive fish covering the different templates and concentrations applied in this study. The samples were selected based on the initial PCR-screening (Table 1), and the results are shown in Fig. 2, Supplementary Fig. 3 and Supplementary Table 1.

### Perfect repair

We aimed to analyze whether any of the repair templates performed better with respect to both accurate repair and integration efficiency. We did not see a significant difference between the ODNs when used at the highest concentration (1.5 µM), although the individual variation was large. Average perfect reads were detected as follows: 6.7% (std = 9.4), 3.0% (std = 2.9), 6.7% (std = 6.7), 5.0% (std = 3.7) and 4.4% (std = 3.3) for S 24, S 48, S 84, AS 24 and ds 24 ODNs, respectively (Fig. 2a). Two fish from each of the S 24 and S 84 groups showed very high rate of perfect integration, displaying 18.7, 26.7, 14.1 and 26.2% perfect HDR, respectively. An example of an alignment (sample 14, S 24 ODN) is shown in Supplementary Fig. 4.

The lowest ODN concentration (0.15 µM, tested for AS 24) resulted in only 0.6% average perfect HDR. This was substantially lower than the results obtained for the highest ODN concentration (1.5 µM), with average perfect reads ranging from 3.0–6.7% (Fig. 2a). Likewise, the plasmid template displayed very low integration efficiency and no reads containing FLAG could be detected following the standard read sequence filtering (Methods and Supplementary Fig. 5). However, when we analyzed the raw material (prior to filtering), perfect FLAG sequences were detected, but only in <0.01% of the total reads.

### Erroneous repair

**Figure 3:**
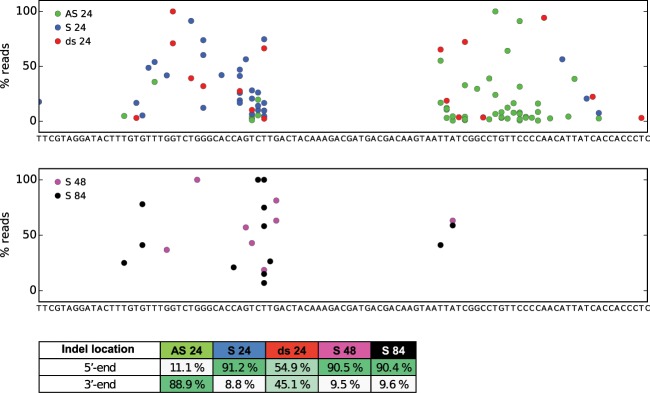
Analysis of indel locations. All the sequence variants were extracted from the group called “Perfect FLAG + indels”. The sequences were aligned to the reference gene containing the inserted sequence. The read count for each indel-containing variant was then converted to percentage of the total read count of variants from the “Perfect FLAG + indels” for all individuals. The percentages were plotted on the reference sequence as colored dots **(a)** AS 24 ODN (green), S 24 ODN (blue) and ds 24 ODN (red). **(b)** S 48 ODN (pink) and S 84 ODN (black). In order to analyze the difference in indel positions between the different groups, the percentages of indels located either at the 5′- or 3′-end of the inserted sequence was calculated for each group and is shown in **(c)**.

**Figure 4:**
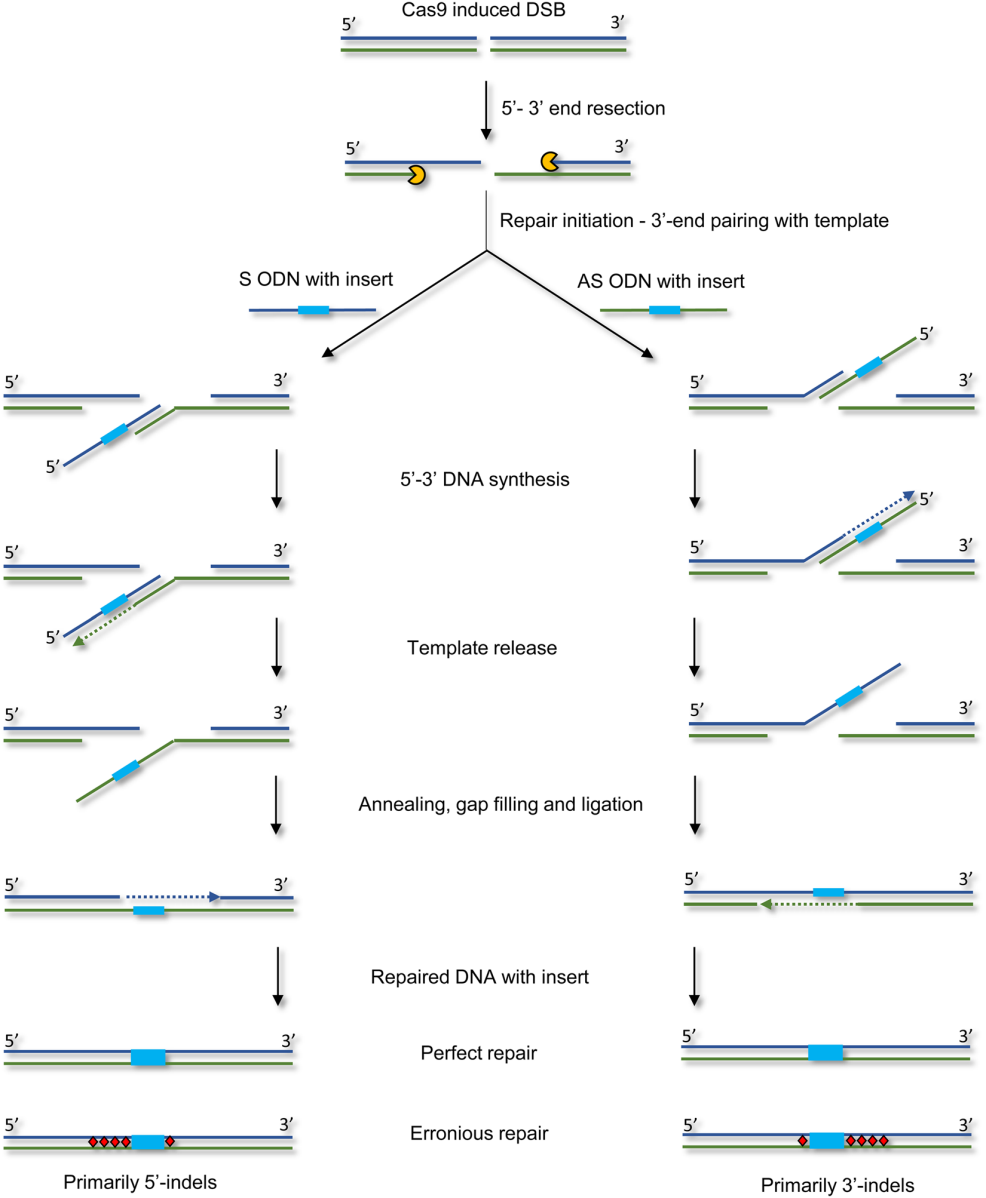
Steps in SDSA and ODN-determined location of the indels. SDSA repair proceeds via distinct steps, starting with resection to yield 3′ overhangs on both sides of the Cas9 induced DSB. The 3′ overhangs pair with the ODN templates and are extended by DNA synthesis copying template sequences. Bridging of the DSB is completed when the newly synthesized strands withdraw from the donor and anneal back at the locus. This results in both perfect and erroneous repair, with indels primarily on the 5′ or 3′-end, depending on the polarity of the ODN. The FLAG insert is shown in light blue, and indels are shown as red diamonds.

In addition to perfect HDR, we detected several imperfect HDR variants with a correct FLAG insert, but various indels within the homology arms. These reads are referred to as “Perfect FLAG + indels” (Fig. 2b). Interestingly, the type of ODN (S, AS or ds) strongly determined the location of these indels (Fig. 3). When using the AS 24 ODN, 88.9% of the indels were located on the 3′-side of the insert. Similar, when using the S 24, 48 and 84 ODNs > 90% of the indels ended up on the 5′-side of the insert. Using the dsODN the indels were equally distributed on the 5′- and 3′-sides of the insert. Based on these results, we suggest that ODN-mediated HDR initiates the SDSA repair pathway. SDSA repair proceeds via distinct steps, starting with resection to yield 3′ overhangs on both sides of the DSB. The 3′ overhangs pair with the ODN template and are extended by DNA synthesis copying template sequences. Bridging of the DSB is completed when the newly synthesized strands withdraw from the donor and anneal back at the locus^47^. An outline showing the steps in this pathway and ODN-determined location of the indels is shown in Fig. 4.

## Discussion

Here, we aimed to establish an efficient method for controlled KI of a FLAG element in F0 salmon using CRISPR/Cas9 and a symmetrical DNA repair template (Fig. 1). To explore the method we tested different templates, concentrations, homology arm lengths and strand complementary (summarized in Table 1). The initial PCR screening revealed a striking difference between the ODNs and the plasmid template, and the ODNs were by far the most efficient. In order to obtain in-depth information about the level of mosaicism and the nature of the integration, we deep-sequenced 76 FLAG-positive fish. We detected average perfect reads within the range of 3.0–6.7%, but the individual variation was large, and some fish displayed perfect repair rates up to 27%. To our knowledge, none have reported this level of perfect HDR in F0 fish. An important aspect hindering a direct comparison with several previous studies is the different methods used to evaluate the HDR efficiency, such as PCR- or restriction-assays, sequencing of a limited number of clones, genotyping based on high-resolution melting analysis, fluorescence etc.^37–40,42,44,46^. Only three studies in zebrafish have used NGS, showing lower perfect repair rates of 1–4%^41^, 1.7–3.5%^45^ and <1%^43^.

Comparing the different repair templates with respect to perfect repair and integration efficiency, our results showed no significant difference between 24, 48 and 84 bp homology using the S ODN. This is in contrast to Boel *et al*.^41^ who reported homology arm length to be the most influential factor and 60 bp homology arms to be the optimal length for symmetrical templates. Moreover, no apparent difference was detected between, ss- and dsODNs (when used at 1.5 µM), which is in contrast to previous findings in *Drosophila*^48^. Our results indicate that ss- vs. dsDNA template is not the main reason for the observed difference in efficiency between the plasmid and ODNs. We hypothesized that the result could be explained by the fact that the plasmid was injected in a substantially lower molar concentration (10 ng/µl is equivalent to 0.004 µM) than the ODNs (1.5 µM). Taken together, these results suggest a concentration dependent mechanism for ODN-mediated HDR in salmon embryos. Likewise, it has been reported that a 0.7 kb insert generated 75% edits when injected at 0.5pmol/l, but only 9% edits when injected at 0.1 pmol/l, in *C. elegans*^49^. Ideally, we would have liked to test a range of different concentrations for all the templates in our study, but this has not been feasible. Although salmon experiments face several challenges in terms of availability in material and slow development, we still believe it is crucial to test out HDR in salmon, as the outcome of the method seems to vary in different species. We hypothesize that the cold rearing temperature and slow development of Atlantic salmon may be an advantage in the context of HDR, allowing a longer timeframe for the integration to occur. The early ontogeny of Atlantic salmon has been described in detail by Gorodilov^50^, who showed how the duration of the developmental stages from fertilization is dependent on temperature. For example, if the eggs are kept at 6 °C, it takes about three months until hatching, in stark contrast to two days for zebrafish. Interestingly, it has been reported that cold shock-treatment increases the frequency of HDR gene editing in induced pluripotent stem cells^51^.

In addition to perfect reads, we detected several reads showing erroneous repair. These reads contained the FLAG insert, but also indels within the homology arms. Most interestingly, we found that the location of these indels were strongly dependent on the polarity of the ODNs. Using the AS ODN 89% of the indels were located on the 3′-side and using the S ODN 90% of the indels were located on the 5′-side. Similarly, when using the dsODN the indels were equally distributed on the 5′- and 3′-sides of the insert, indicating that the repair machinery has no preference regarding the template polarity (S vs. AS ODN). This, in combination with the similarity of the inserts with the ODN template sequence, also strongly indicates that DSB repair using ODNs initiates the SDSA pathway, as previously suggested for *C. elegans*^49^ and zebrafish^41^. Our findings suggest that the 3′-end pairing with the template and initial DNA synthesis occur with high fidelity, while the steps involving annealing, gap filling, and ligation are more prone to errors. The cause of these errors is unclear, but various mechanisms of template switching have been suggested^41^. Our data supports the template switching theory, as the origin of the inserts predominantly have high similarity with the ODN template sequence (Supplementary Fig. 6). To our knowledge, the ODN (S vs. AS) dependent location of indels has not been reported by others. We suggest taking this information into account when designing ODN repair templates for HDR. To obtain a high rate of in frame integration 5′- end indels must be avoided, making AS ODNs the preferred template.

We have in this study observed that ODNs (S, AS and ds) with 24, 48 and 84 bp homology arms integrates perfectly at a relatively high rate (up to 27%) into salmon embryos. These results are obtained from sequencing of DNA from fin clips, which might not perfectly reflect germline transmission efficiency. However, considering the high fecundity of salmon females (8000–10000 eggs), a potential quick integration into broodstock is possible by crossing F0s. For example, if parental F0 fish have 15% perfect integration, crosses will produce ~180–225 F1 offspring with double allelic KI. To increase the efficiency further studies could focus on the concentration of ODN template, as this clearly affects the efficiency of integration (Fig. 2). However, focus could also be aimed at Cas9. Currently we are using Cas9 mRNA, this probably results in more variants compared to Cas9 protein as observed previously^52^. Unfortunately, although we have performed multiple trials with Cas9 protein, we have not yet been able to successfully use it in salmon embryos. It is also possible to explore other nucleases^53^ to improve efficiency and accuracy of the CRISPR KI protocol. Another possibility would be to use short-life Cas9 variants, which have been reported to reduce toxicity and off-target editing^54,55^.

A challenge with the ODN technology is the possibility to make these ODNs long enough for, for example full gene integration. While synthesis of ODNs were previously restricted to a maximum length of <200 nucleotides, recent technologies now allow generation of longer sequences^56,57^, and simple ssDNA synthesis over 10 kb using asymmetric PCR has been demonstrated^58^. Commercial manufacturers also offer synthesis of long ssDNA, although at a relatively high cost. Nevertheless, this enables the insertion of longer sequences such as reporters, gene tags, regulatory elements or even genes. However, editing efficiency is sensitive to insert size, elegantly shown by Paix and colleagues by taking advantage of the split-GFP system^47^.

We have compared various DNA repair templates for HDR in salmon, and our results show that ODN templates induce highly efficient HDR integration at the target site, much higher than previously observed in any fish species. Our results also indicate that the integration occurs via the SDSA repair pathway and is dependent on template concentration. Interestingly our data also gives further clues to how the SDSA repair pathway may work, as we for the first time in any species show in detail that the distribution of indels is dependent on ODN polarity.

## Methods

### Ethics statement

This experiment was approved by the Norwegian Animal Research Authority (NARA, permit number 5741) and the use of these experimental animals was in accordance with the Norwegian Animal Welfare Act.

### Preparation of Cas9 RNA and gRNA

The *slc45a2* CRISPR target sequence is described in Edvardsen *et al*.^9^. The target sequence was blasted against the reference genome of salmon and show no other hits than to the gene in question. Preparation of gRNA and *cas9* mRNA was performed as previously described^9^ with the following exceptions: for *in vitro* transcription of gRNA we used the HighScribe T7 Quick High Yield RNA Synthesis Kit (NEB) according to the protocol for short transcripts. The RNeasy MiniKit spin column (Qiagen) was used to purify the gRNA.

### Design and preparation of donor DNA templates for *slc45a2*

S- and AS ODNs were ordered from Integrated DNA Technologies (Leuven, Belgium). They were designed by copying 24/48/84 nucleotides on each side of the CRISPR cut-site, with a 29 bp insert comprised of TT-FLAG-TAA. TT was included to keep the open reading frame of FLAG, and the STOP codon (TAA) was added to ensure an albino phenotype for *slc45a2* CRISPR mutants, regardless of a successful KI-event. Aiming to compare ss- vs. dsDNA, we prepared a dsODN (with 24 bp homology arms) by annealing S and AS. The design is illustrated in Fig. 1. Another pair of S and AS ODNs (24 bp homology arms) were designed with the purpose of cloning into a plasmid (pCR^TM^4-TOPO vector). The design is identical to the one described above, with the addition of gRNA target sequences on each side for *in vivo* release of the template and A-overhangs in the 3′ends. The S and AS ODNs were annealed, and cloning performed according to the TOPO® TA Cloning® Kit for Sequencing. The different repair templates are described in Table 2.

**Table 2:** Description of the different repair templates used. *All repair templates were symmetrical, with both left and right homology arms of the same length. **The polarity of the ssODNs are relative to *slc45a2*.

Repair template name	Repair template characteristics			
	Template	Homology arm length (bp)*	ss/ds DNA	Polarity**
Plasmid 24	Plasmid	24	ds	
S 24 ODN	ODN	24	ss	S
AS 24 ODN	ODN	24	ss	AS
ds 24 ODN	ODN	24	ds	
S 48 ODN	ODN	48	ss	S
S 84 ODN	ODN	84	ss	S

### Microinjection

Salmon eggs and sperm were delivered by Aquagen (Trondheim, Norway). Fertilization and microinjections were carried out as described previously^9^ using 50 ng/µl gRNA and 150 ng/µl *cas9* mRNA in nuclease free water and a FemtoJet®4i (Eppendorf) microinjector. The ODNs (S, AS or ds) were added to the injection mix with a final concentration of 1.5 or 0.15 µM, and the plasmid with a final concentration of 2.5 or 10 ng/µl (corresponding to 0.001 and 0.004 µM, respectively).

### Analysis of mutants

When kept at 6–8 °C, the salmon eggs will hatch approximately three months post fertilization. The *slc45a2* CRISPR mutants are easily recognized in newly hatched embryos and in juveniles, due to the lack of pigment, and these individuals (albinos) were selected for further DNA analyses. DNA was extracted from caudal fins using DNeasy Blood & Tissue kit (Qiagen). To ensure complete homogenization, the tissue was incubated overnight at 56 °C using a thermomixer. DNA was eluted in 30 µl nuclease free water. To identify FLAG-positive mutants PCR was performed on genomic DNA, with the forward primer targeting the FLAG-sequence (5′-CTACAAAGACGATGACGAC) and the reverse primer targeting *slc45a2* (5′-CGCAACGACTACACATTAT). The PCR-products were evaluated on 1% agarose gels. In order to verify insertion of FLAG and to assess the level of mosaicism, a fragment covering the entire target site was amplified in selected samples (n = 76) with a two-step fusion PCR to prepare for sequencing by Illumina MiSeq, as described in^30^. The following primer sequences were used in the first PCR-step; 5′-tctttccctacacgacgctcttccgatctCAGATGTCCAGAGGCTGCTGCT and 5′-tggagttcagacgtgtgctcttccgatctTGCCACAGCCTCAGAATGTACA (gene specific sequence indicated in capital letters).

### Analysis of MiSeq data

Fastq files were filtered and trimmed with Cutadapt^59^, and variants were called using a custom script (Supplementary Fig. 5). Finally, read counts were reported for the variants containing the inserted sequence, separating those with a perfect match to the entire target sequence (referred to as perfect HDR), and those with a correct insert sequence but various mismatches in the rest of the target sequence (referred to as perfect FLAG + indels) (Fig. 2). In addition, read counts were reported for variants containing degenerated insert sequences (≥50% intact insert sequence, referred to as degenerated FLAG), and wild type sequences (Supplementary Fig. 3).

### Analysis of indel locations in the “Perfect FLAG + indels” group

All the sequence variants were extracted (after filtration in the previous variant analysis) from the group called “Perfect FLAG + indels”. Using Geneious, the sequences were aligned to the reference gene containing the inserted sequence using the “Highest Sensitivity” option. The alignment was used to extract the information about indel positions, and for each deletion the location of the 5′ end of the deleted sequence was chosen to represent the position. The read count for each indel-containing variant was converted to percentage of the total read count of variants from the category “Perfect FLAG” for all individuals. The percentages were plotted on the reference sequence with colors showing AS 24 (green), S 24 (blue), ds 24 (red), S 48 (pink) and S 84 (black) (Fig. 3). In order to analyze the variation in indel positions between the different templates, the percentages of indels located either at the 5′- or 3′-side of the inserted sequence was calculated for each group.

### Statistical analyses

D’Agostino Person normality test (column statistics) were used to asses normal distribution of the data. Non-parametric statistical analyses were performed using a Kruskall-Wallis test, followed by Dunn’s multiple comparison test. The tests were carried out using GraphPad Prism 8.0.1.

## Supplementary information

Supplementary information

## Data Availability

Data generated or analyzed during this study are included in this article (and its Supplementary Information File).
