# Supplementary material for: Indel locations are determined by template polarity in highly efficient in vivo CRISPR/Cas9-mediated HDR in Atlantic salmon
Source: Sci Rep. 2020 Jan 15;10:409. doi: 10.1038/s41598-019-57295-w (PMC6962318; doi:10.1038/s41598-019-57295-w)
Supplement: Supplementary file 1 — Supplementary information [file 41598_2019_57295_MOESM1_ESM.pdf]

Supplementary Figures and Supplementary Table:

Indel locations are determined by template polarity in highly efficient *in vivo* CRISPR/Cas9-mediated HDR in Atlantic salmon

Authors: Anne Hege Straume[1], Erik Kjærner-Semb[1], Kai Ove Skaftnesmo[1], Hilal Guralp[1], Lene Kleppe[1], Anna Wargelius[1], Rolf Brudvik Edvardsen[1]

Affiliations: [1]Institute of Marine Research, P.O. Box 1870, Nordnes, NO-5817, Bergen, Norway

E-mail: rolfbe@hi.no

Supplementary Figure 1. Sorting of *scl45a2* mutants

A

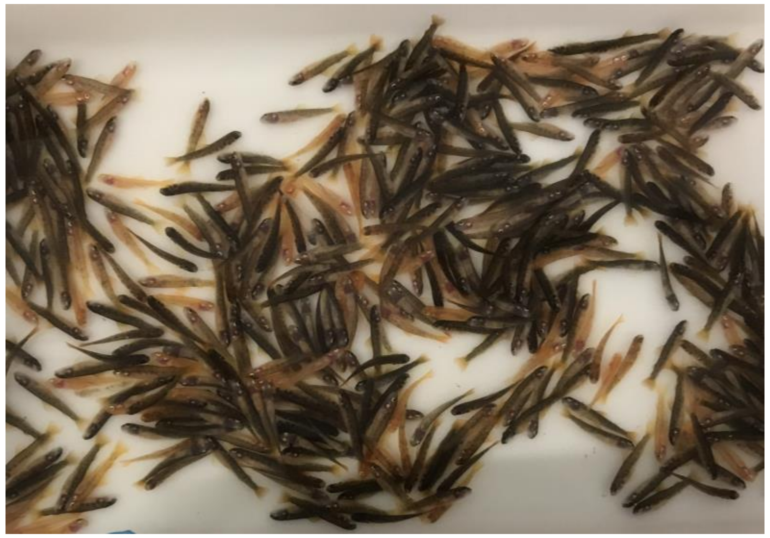

The *slc45a2* CRISPR mutants are easily recognized in newly hatched embryos and in juveniles, due to the lack of pigment, and only these individuals (albinos) were picked for further DNA analyses. Juveniles injected with 10 ng/ul plasmid is shown in **A** (prior to sorting), and **B** (after sorting of albinos). Juveniles injected with S ODN, dsODN and AS ODN is shown after sorting of albinos in **C, D** and **E**, respectively.

B

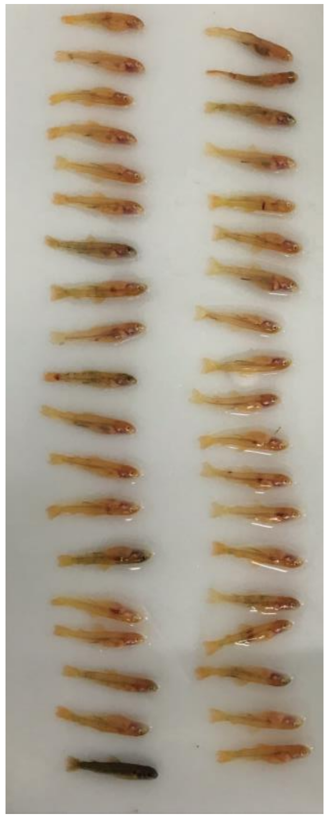

C

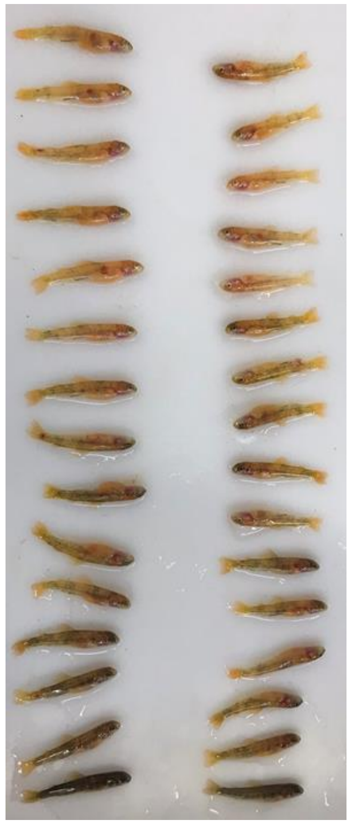

D

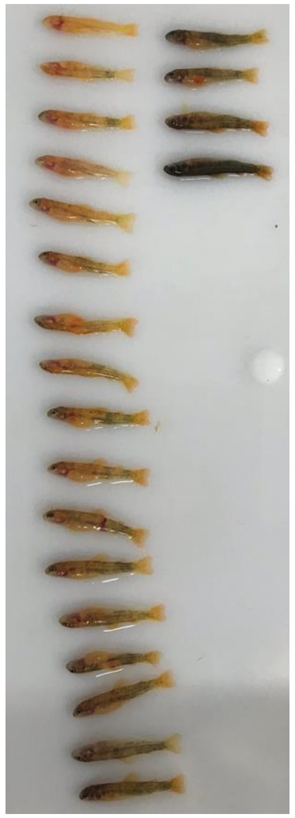

E

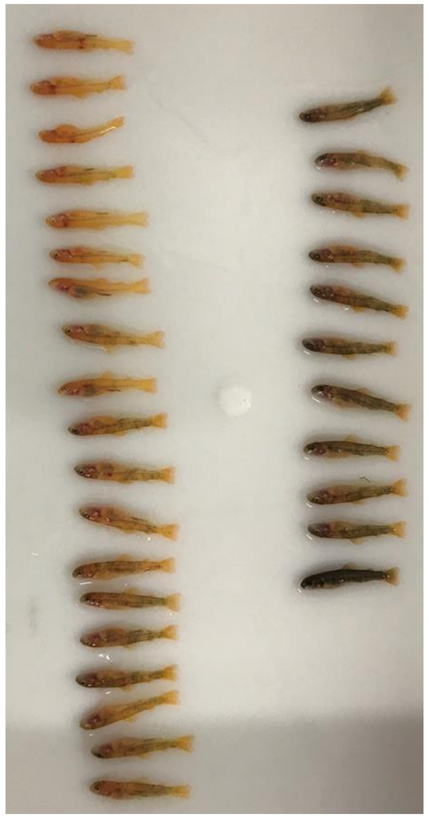

Supplementary Figure 2. Example of PCR screening for FLAG positive mutants

A:

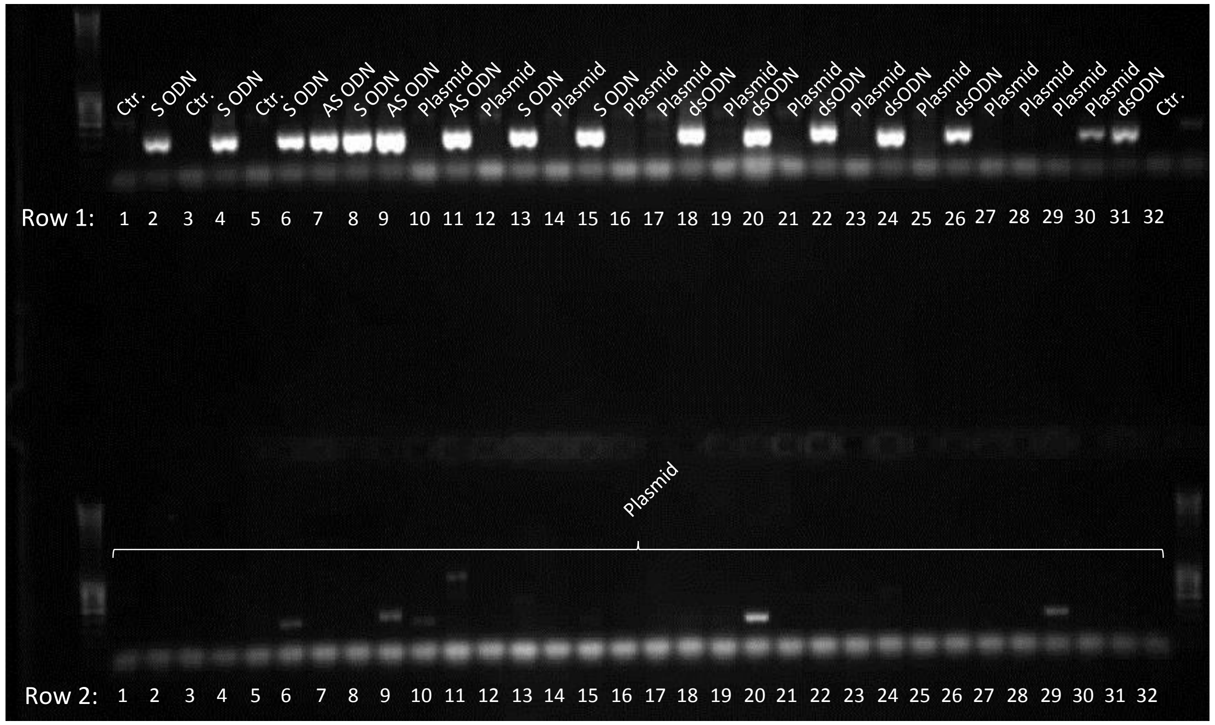

A: PCR and 1% agarose gel electrophoresis were performed to identify FLAG-positive *slc45a2* CRISPR mutants. In this example screening was performed with embryos injected with S, AS and dsODNs, and plasmid with 24 bp homology arms. **B**: The original uncropped gel picture

B:

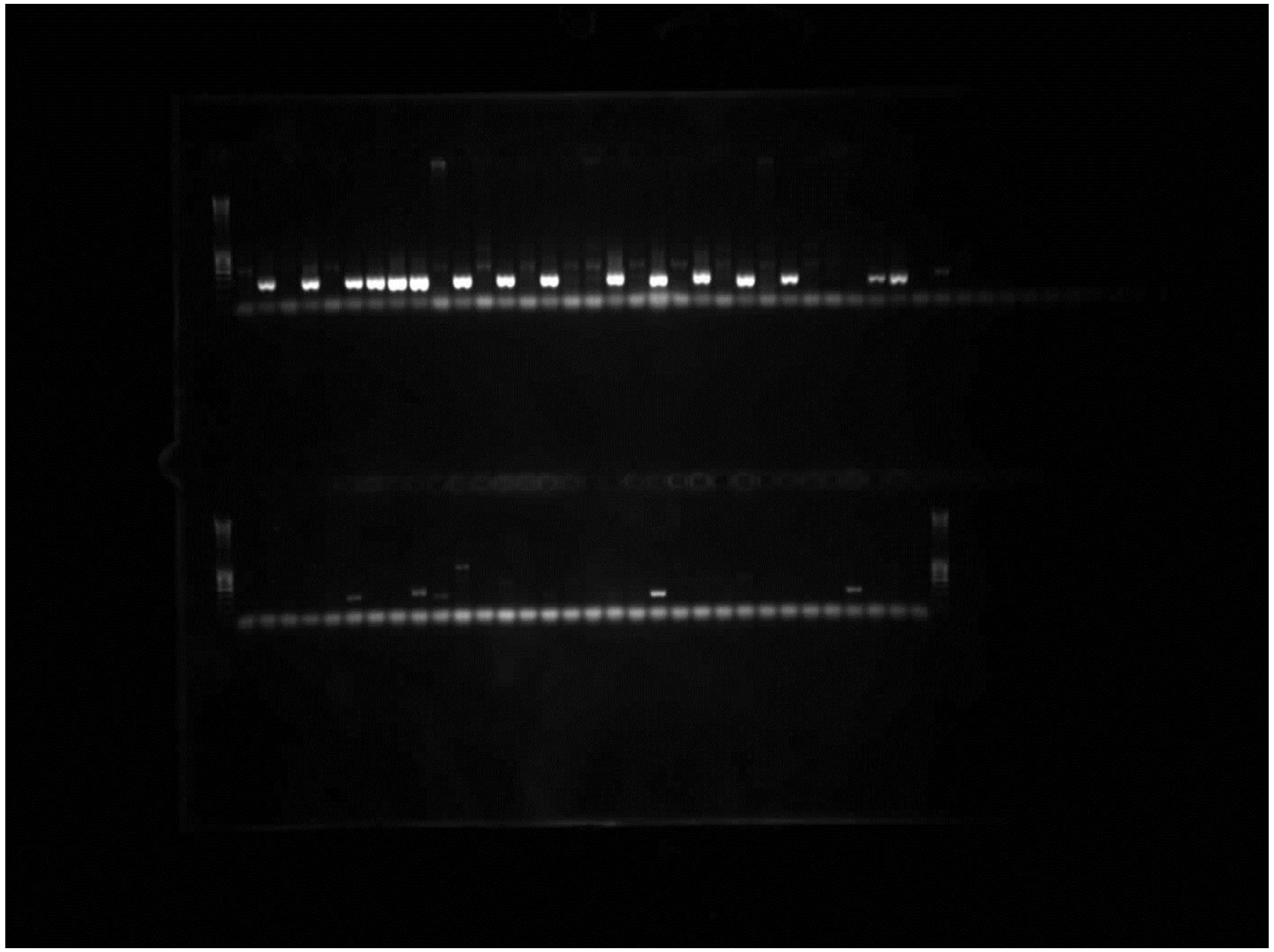

Supplementary Figure 3. Degenerated and wild type reads

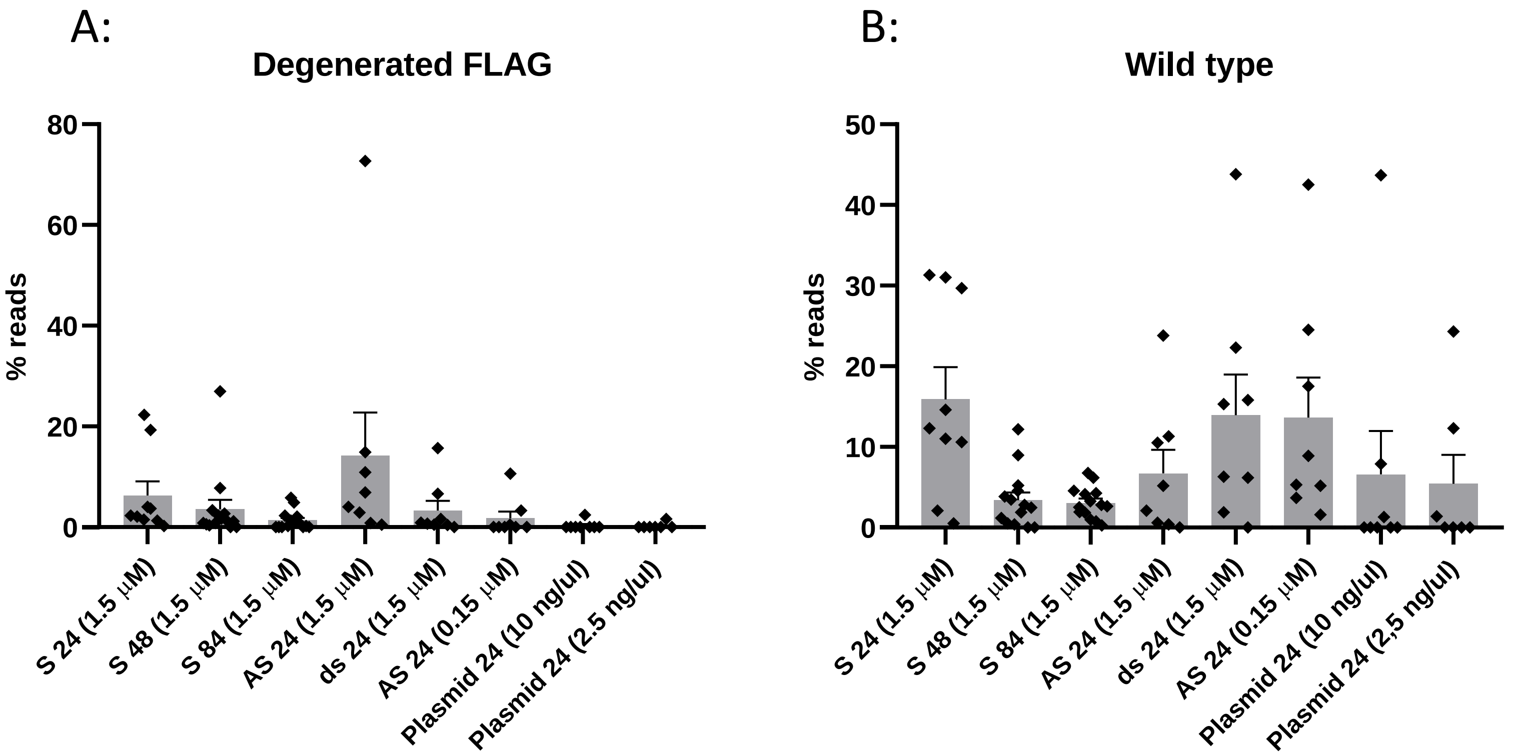

A fragment covering the entire CRISPR target site was amplified (n = 76) prior to Illumina MiSeq. **A: Degenerated FLAG** (reads with mismatches in the insert sequence, but ≥ 50 % of the insert sequence is intact) and **B: Wild type.** Reads displaying **Perfect HDR** and **Perfect FLAG + indels** is shown in Figure 2. Read counts for each group are given in % of the total number of reads with at least 100 identical reads, for each sample. Individual samples are represented by black diamonds, and grouped for each of the different repair templates, at different concentrations (represented by grey bars). The error bars indicate the SEM of the mean for each group.

Supplementary Figure 4. Example of NGS reads alignment

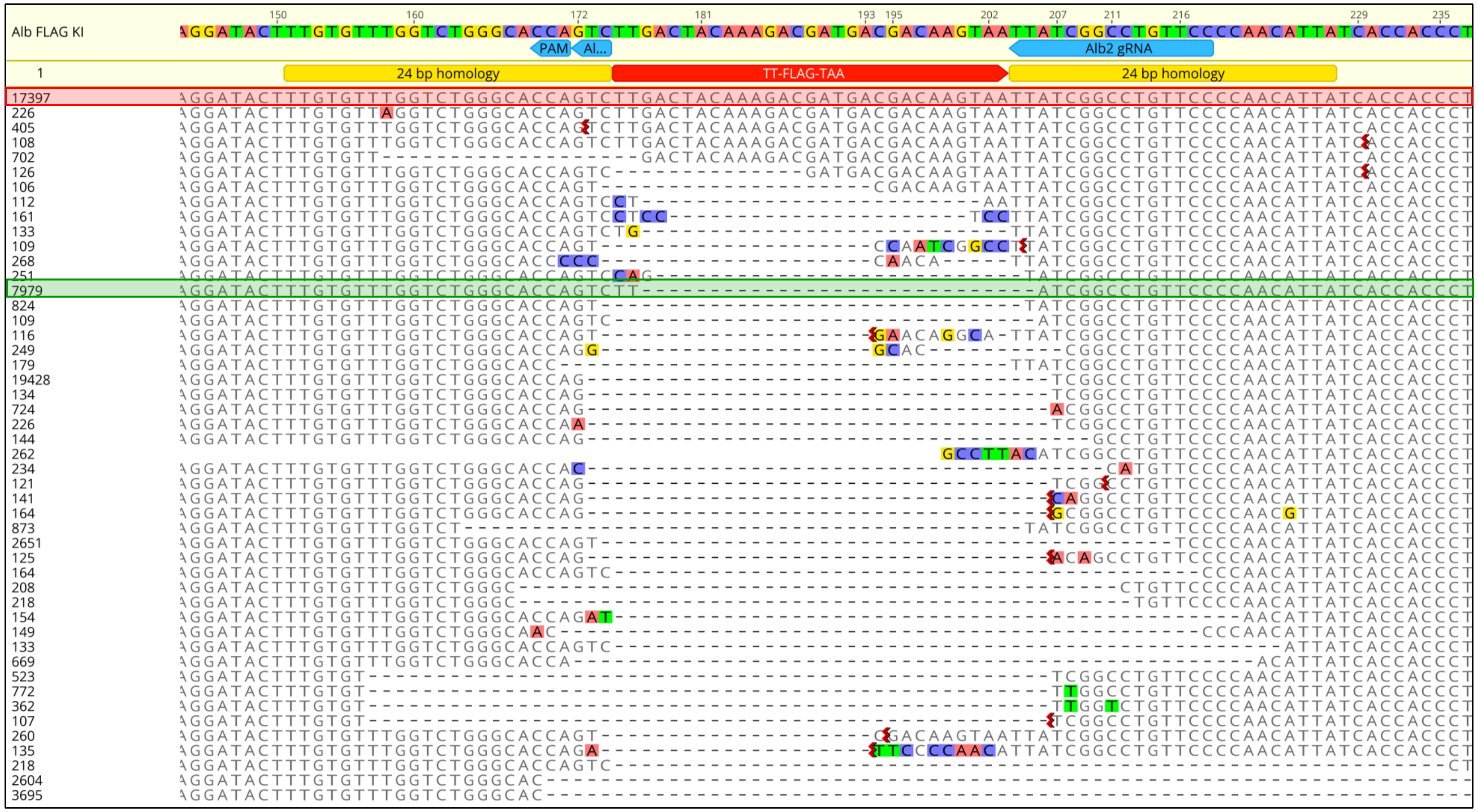

Genious alignment of reads reported from sample 14 (Repair template: S 24 ODN) displaying 26,7 % perfect HDR. The column with numbers to the left is the number of reads of each sequence. In this example, 17397 reads display perfect HDR (highlighted with red), and 7979 reads display wild type (highlighted with green).

Supplementary Figure 5. NGS analysis workflow

1) Fastq files were filtered and trimmed with cutadapt (ref) using the following specifications:

↓

Two bases were removed from 5' and 3' ends

↓

Bases with base quality < 25 were removed from the 3' end

↓

Minimum read length was set to 100 bp after trimming

↓

2) Variants were called using a custom script (suppl.) with the following specifications:

↓

Used only position 20-200 in the reads (forces minimum read length to 200 bp)

↓

If base quality was < 20 → converted bases to N's

↓

Maximum 5 N's were allowed per read

↓

Identical reads were grouped (referred to as variants)

↓

Retained only variants with a BLAST match against the target sequence (E-value < 1E-03)

↓

Variants were grouped if they only differed by maximum 5 N's as long as none of the variants differed by any other nucleotides

↓

For each group, the variant with the least N's was chosen as representative

↓

Retained only variants covered by a minimum of 100 identical reads

↓

Variants were grouped if they only differed by maximum 5 N's as long as none of the variants differed by any other nucleotides

Illustration of the settings applied for filtering, trimming and variant calling of the MiSeq reads. Fastq files were filtered and trimmed with cutadapt (ref) using the following specifications; two bases were removed from 5' and 3' ends, bases with base quality < 25 were removed from the 3' end and minimum read length was set to 100 bp after trimming. Variants were then called using a custom script. We only used positions 20-200 in the forward strand reads (forces minimum read length 200 bp). All bases with base quality < 20 were converted to N's, and maximum 5 N's were allowed per read. Identical reads were then grouped (referred to as variants) and we only retained variants with a BLAST (ref) match against the target sequence (E-value < 1E-03) → variants that were only differing by up to 5 N's were grouped as long as none of the variants differed by any nucleotides → for each group the variant with the least N's was chosen as representative → only retained variants supported by a minimum of 100 reads → variants were grouped if they differed by up to 5 N's as long as none of the variants differed by any nucleotides. Finally, read counts were reported for the variants containing the inserted sequence, separating those with a perfect match to the entire target sequence, and those with a correct insert sequence, but mismatches in the rest of the target sequence. In addition, read counts were reported for variants containing degenerated insert sequences (≥ 50% intact insert sequence), as well as for wild type sequences.

Supplementary Figure 6. Alignment of insert sequences

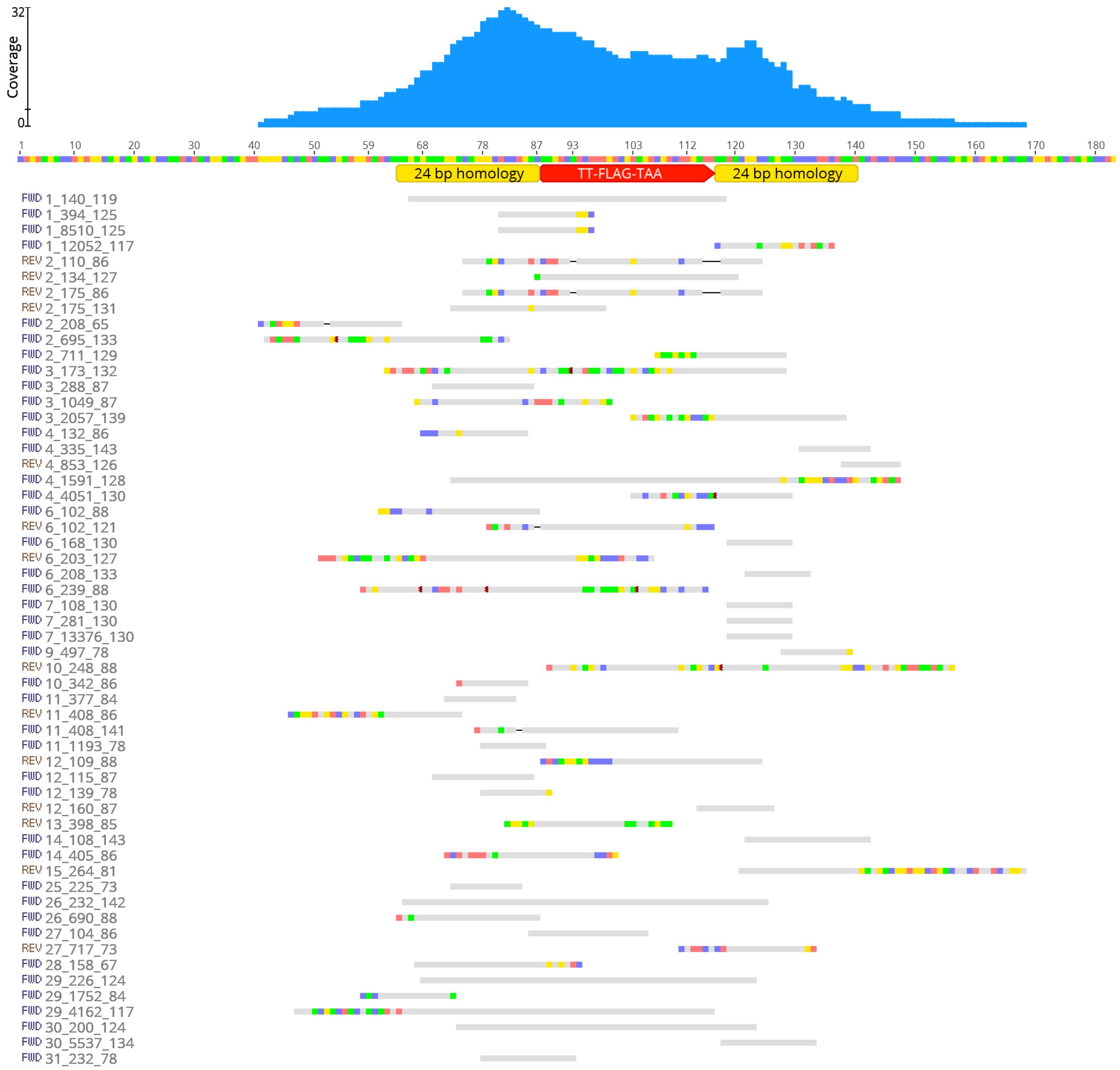

Origin of the inserts from the 24 bp homology arms template groups. 56 out of 62 insertions (≥ 10 bp) maps to and have high similarity with the ODN template sequence. Grey bars indicate identical sequence, while colors indicate mismatches. The column with numbers to the left shows: sample number_read counts_insert position.

Supplementary Table 1**:** Comparison of HDR accuracy among different templates and concentrations, analyzed by MiSeq.

Concentration	Perfect HDR (%)	Perfect FLAG + indels (%)	Degenerated FLAG (%)	Other edits (%)	Wild type (%)
1.5 µM	8.63	10.88	4	71.25	5.24
1.5 µM	4.93	1.67	0.8	92.23	0.37
1.5 µM	10.33	5.37	10.9	72.84	0.56
1.5 µM	6.87	4.7	14.9	71.47	2.06
1.5 µM	0	0.27	72.7	27.03	0
1.5 µM	2.12	1.6	6.9	78.12	11.26
1.5 µM	5.96	14.95	2.9	65.73	10.46
1.5 µM	0.85	1.09	0.5	73.78	23.78
1.5 µM	4.61	0.72	2.1	78.01	14.56
1.5 µM	1.24	8.62	1.5	86.54	2.1
1.5 µM	0.79	5.79	19.3	73.63	0.49
1.5 µM	18.66	1.74	3.7	64.93	10.97
1.5 µM	5.15	1.44	2.3	80.47	10.64
1.5 µM	26.7	2.21	1.3	57.54	12.25
1.5 µM	1.32	0.64	22.3	44.41	31.33
1.5 µM	1.59	1.85	0.2	65.39	30.97
1.5 µM	0.54	2.91	4	62.90	29.65
2.5 ng/µl	0	0	0	98.60	1.4
2.5 ng/µl	0	0	0	100.00	0
2.5 ng/µl	0	0	0	75.70	24.3
2.5 ng/µl	0	0	1.6	98.40	0
2.5 ng/µl	0	0	0	87.70	12.3
2.5 ng/µl	0	0.28	0	99.72	0
2.5 ng/µl	0	0	0	100.00	0
1.5 µM	3.18	0.45	0.9	80.17	15.3
1.5 µM	9.41	1.55	0.4	66.34	22.3
1.5 µM	2	1.46	0.5	89.74	6.3

1.5 µM	0	11.57	15.7	72.73	0
1.5 µM	6	9.71	6.6	75.79	1.9
1.5 µM	8.72	19.5	1.6	63.98	6.2
1.5 µM	2.63	1.78	0.7	79.09	15.8
1.5 µM	3.14	0	0	53.06	43.8
10 ng/µl	0	0	0	100.00	0
10 ng/µl	0	0	0	100.00	0
10 ng/µl	0	0	0	100.00	0
10 ng/µl	0	0	0	100.00	0
10 ng/µl	0	0	2.4	97.60	0
10 ng/µl	0	0	0	92.10	7.9
10 ng/µl	0	0	0	98.70	1.3
10 ng/µl	0	0	0	56.30	43.7
0.15 µM	0	0	0	91.07	8.93
0.15 µM	0	0	0	96.30	3.7
0.15 µM	0.79	0.73	0.5	96.34	1.64
0.15 µM	1.5	0	0	93.22	5.28
0.15 µM	0.97	0	10.6	83.26	5.17
0.15 µM	1.01	0	3.3	71.18	24.51
0.15 µM	0.64	0	0	56.86	42.5
0.15 µM	0	0	0	82.50	17.5
1.5 µM	2.67	0.77	1.16	94.88	0.52
1.5 µM	0.9	0	2.72	95.19	1.19
1.5 µM	8.87	0.2	0.38	87.06	3.49
1.5 µM	1.22	0	0.86	96.03	1.89
1.5 µM	6.03	0	0.58	93.39	0
1.5 µM	1.5	0	0	93.93	4.57
1.5 µM	0.6	0	0	99.40	0
1.5 µM	0.84	1.35	0.8	94.52	2.49
1.5 µM	2.51	0.59	7.78	88.74	0.38
1.5 µM	8.85	0	1.67	86.65	2.83
1.5 µM	1.73	0.24	1.14	87.91	8.98

1.5 µM	3.21	1.64	2.31	87.62	5.22
1.5 µM	0.2	0	26.96	68.97	3.87
1.5 µM	2.39	14.54	3.36	67.51	12.2
1.5 µM	3.42	0.77	2.27	91.62	1.92
1.5 µM	6.39	1.99	0.72	88.10	2.8
1.5 µM	14.12	1.92	0	83.70	0.26
1.5 µM	5.99	2.07	2.07	88.02	1.85
1.5 µM	1.05	0.21	4.91	91.17	2.66
1.5 µM	6.68	6.39	0.22	84.18	2.53
1.5 µM	3.36	0	0	90.45	6.19
1.5 µM	26.22	10.52	0	62.30	0.96
1.5 µM	3.13	0.64	0	92.94	3.29
1.5 µM	10.36	0.18	0.22	88.47	0.77
1.5 µM	4.35	2.12	1.25	87.72	4.56
1.5 µM	4.45	2.87	0	85.89	6.79
1.5 µM	0.63	0	5.85	89.26	4.26
1.5 µM	2.98	0.29	1.32	91.25	4.16
